# Botanical biopesticides have an influence on tomato quality through pest control and are cost-effective for farmers in developing countries

**DOI:** 10.1371/journal.pone.0294775

**Published:** 2023-11-28

**Authors:** Waheed Akhter, Farhan Mahmood Shah, Minglu Yang, Shoaib Freed, Muhammad Razaq, Angela Gerald Mkindi, Hina Akram, Abid Ali, Khalid Mahmood, Muhammad Hanif

**Affiliations:** 1 Department of Entomology, Faculty of Agricultural Sciences and Technology, Bahauddin Zakariya University, Multan, Punjab, Pakistan; 2 Key Laboratory of Integrated Pest Management on Crops in Southern Xinjiang, College of Agriculture, Tarim University, Alar, Xinjiang, China; 3 Department of Sustainable Agriculture, Biodiversity and Ecosystems Management, The Nelson Mandela African Institution of Science and Technology, Arusha, Tanzania; 4 Department of Pharmaceutics, Faculty of Pharmacy, Bahauddin Zakariya University, Multan, Punjab, Pakistan; 5 College of Life Science, Shenyang Normal University, Shenyang, China; 6 Department of Entomology, Faculty of Agriculture, University of Agriculture, Faisalabad, Punjab, Pakistan; 7 Institute of Chemical Sciences, Bahauddin Zakariya University, Multan, Punjab, Pakistan; University of Carthage, TUNISIA

## Abstract

Synthetic insecticides heavily applied to manage agricultural pests are highly hazardous to the environment and non-target organisms. Their overuse through repeated treatments in smallholder farming communities is frequent. Botanical biopesticides are ideal for sustainable pest management in agricultural environments by keeping synthetic insecticide use at a minimum. Here we evaluated a locally prepared neem seed extract (NSE) alongside emamectin benzoate against both lepidopteran pests *Helicoverpa armigera* (Hübner) and *Spodoptera exigua* (Hübner) on tomato *Lycopersicon esculentum* Mill under natural field conditions in Pakistan. We compared pest severity, fruit injury, quality, marketability, and cost:benefit ratio (CBR) between treatments. The concentration of azadirachtin A in the NSE was 26.5 ppm. NSE at 2% (20 mL/L) and the emamectin benzoate at the recommended field rate in Pakistan were sprayed weekly throughout the fruiting stage. The pest larvae were significantly more abundant on fruits than on flowers and leaves. Fruit injury and losses were significantly more important in untreated control compared to NSE and emamectin benzoate treatments. NSE efficacy varied with respect to the cultivars used and the seasons. Cultivar Eden harboured more pests than Adventa, and emamectin benzoate suppressed more pest individuals than NSE. Both the insecticidal treatments were comparable in terms of marketable yield productions as well as unmarketable, uninjured, and recovered fruit yields. NSE generated a higher CBR (1: 9.26) than emamectin benzoate (1: 3.23). NSE suppressed pests by acting as an antifeedant, similar to its synthetic counterpart. Smallholder growers can thus use NSE as a cost-effective solution in tomato pest management in Pakistan.

## Introduction

Insect pests pose severe risks to agriculture and food worldwide. Interventions used to control insect pests include both prophylactic and curative methods. One curative method is applying insecticides repeatedly for effective management of insect pests, which is typically more common among vegetable growers [1]. The avermectin Emamectin benzoate is a novel macrocyclic lactone bioinsecticide, developed for integrated pest management (IPM) against lepidopterans infesting field crops. It affects arthropod nervous system by increasing chloride ion flux at the neuromuscular junction, causing feeding cessation and irreversible paralysis. It is highly active against lepidopteran pests including *Helicoverpa armigera* (Hübner), *Spodoptera exigua* (Hübner), *Plutella xylostella* (L.), and *Trichopluisa ni* (Hübner) [2, 3]. Repeated long-term overuse of pesticides can select for biological resistance in pest and hamper biocontrol functions through lethal and sublethal exposures [4, 5]. Therefore, alternative strategies that are safe and effective are needed to keep synthetic insecticide use at a minimum, especially in pesticide-dominated pest control systems of lesser developed countries.

Plant-derived natural pesticides can offer a better alternative to synthetic chemical pesticides [6–8]. Botanicals are plant-produced secondary metabolites with strong insecticidal attributes applied as purified compounds or complex mixtures [9]. The biochemical constituents in botanicals are highly target-specific, rapidly biodegradable, and safer for non-target organisms [6, 10]. Due to their complex chemistries and novel modes of action, botanicals can be effective against resistant pests, also slowing the risk of insect developing resistance [11–17]. Botanicals are a top research priority among scientists and policymakers worldwide because they can reduce chemical pesticide use while also making pest management more economical, viable, and sustainable [11, 18–20]. Vegetable production using botanicals can be more cost-effective as demonstrated by a cost:benefit analysis in managing serious vegetable pests in Bangladesh [21] and *Drosophila suzukii* (Matsumura) on berry crops in Italy [22].

Neem oil extracted from seeds of the neem tree (*Azadirachta indica* A. Juss, family Meliaceae) has broad-scale implications against a wide range of agricultural, veterinary, and medical pests [23, 24]. Neem seed extract (NSE) contains at least 100 biologically active compounds, with azadirachtin being the major insecticidal ingredient. Azadirachtin deters feeding, affects hormonal functions in juvenile stages, reduces ecdysone, deregulates growth, alters development and reproduction, and disrupts molting processes. Because of these various attributes, azadirachtin has acquired commercial recognition as a promising biopesticide. Azadirachtin is applied as aqueous, alcoholic and azadirachtin-enriched extracts. The residual activity lasts for 4–8 days post-application. The commercial products are very effective against hemipterans and lepidopterans infesting field crops [14, 19, 25], and there is a potential for production of cost-effective extracts [26]. Hence, in pesticide-dominated pest management systems in developing countries, where pesticide poisoning and residual contamination are increasingly severe [27–29], the use of botanicals can likely reduce chemical pesticide load and concerns.

*Helicoverpa armigera* and *S*. *exigua* are polyphagous pests of cotton, maize, tobacco, grams, pulses, vegetables, ornamentals, and other important crops [30–32]. In successions to multiple hosts, these pests receive exposure to different insecticides and develop resistance [33–35]. Total losses resulting from *H*. *armigera* were estimated at US$ 5 billion, annually. About half of the chemicals among the total used in agriculture are applied to control this pest in China and India [36]. In Australia, resistance in *H*. *armigera* to organochlorines was noted in the late 1960s whereas pyrethroids developed resistance to this pest four years after their start of applications on the cotton crop [37]. Resistance to conventional insecticides and new molecules like avermectins, oxidiazienes and spinosyns has been recorded in India [38]. Resistance in *S*. *exigua* to molecules like spinosad, metaflumizone, chlorantraniliprole, emamectin benzoate or methoxyfenozide has been reported in China [39–41], Brazil [42], the USA [43], and Pakistan [44–46].

This study focused on the important tomato crop, *Lycopersicon esculentum* Mill, which is grown and consumed worldwide. Both *H*. *armigera* and *S*. *exigua* are dominant lepidopteran pests in tomatoes where they can reduce production yield [47] and management relies on pesticides. A tomato crop currently receives about 10–12 applications of emamectin benzoate per season, which equals 150–200 US$/ha, and botanicals are not readily marketed for their applications in the field in Pakistan like many other developing countries. In the exhaustive reviews by Isman and Grieneisen [48] and Benelli et al. [17], it was noted that most of the research studies using botanicals include laboratory bioassays without chemical characterization of the extracts, thus lacking reproducibility and novelty, limiting their commercialization. It was also emphasized that studies in the developing countries should be conducted for utilization of botanical extracts for crop protection in the fields as these will be of more worth than bioassays in the laboratories. Previous research showed a promising effectiveness of NSE on *H*. *armigera* populations, plant growth, infestation of fruits and effect on yield of tomato, compared to synthetic products or other IPM modules [49–53]. Nevertheless, none of these publications reported chemical standardization of used extracts; hence their findings are not comparable and reproducible and could not reach conclusions. To fill this gap, the current study assessed the efficacy and economic viability of NSE with characterization of its chemical composition relative to the synthetic pesticide emamectin benzoate in field trials against *H*. *armigera* and *S*. *exigua*. The current study used internationally recognized fruit quality and grading standards for marketability and assessed whether NSE can offer promise as a cost-effective alternative in tomato pest management.

## Materials and methods

### Study site and seedling preparation

This study was conducted from November to May in the 2014 and 2015 tomato growing seasons at the Agricultural Research Farm of Bahauddin Zakariya University, Multan, in Punjab province of Pakistan (30° 11’ 44” N / 71° 28’ 31” E). Multan is a subtropical region with a winter season from November to February followed by a spring season in the month of March [54]. Many vegetables, cereals and fruit crops are grown in this region and synthetic pesticides are the most common option of pest control. Tomatoes in this region are grown under open field condition for both subsistence and commercial purposes.

Hybrid seeds of tomato cultivars Eden and Adventa (ICI Pakistan Limited, Karachi, Pakistan) were purchased from a local market in Multan. In the second week of October, seeds were sown in the nursery using beds of area measuring 1 m × 1 m. After one month, seedlings were transplanted bare-rooted to one-sided field beds. The interplant distance was 30 cm between seedlings on a row. The beds were 0.5 m wide and 0.75 m apart. The treatment plots were 6 m long with four rows. Treatment plots were separated by a 1 m buffer zone to avoid pesticide spill-over. All agronomic practices were followed according to recommendations by the local research station.

### Experimental setup

The cultivar and insecticidal treatment were the factors evaluated in this study. A factorial experiment arranged in randomized complete block design with three replicates per cultivar-insecticide treatment combination was used in this study. Eden and Adventa cultivars and three treatments (Rider® (Emamectin benzoate 1.9 EC; 80 mL/ha, Suncrop Limited, Multan, Pakistan); neem seed extract (NSE; 20 mL/L water); and an untreated control (no spray)) were evaluated for their effectiveness against *H*. *armigera* and *S*. *exigua* alongside crop damage and infestation severity. Pesticide applications were started at weekly intervals at the beginning of the fruit formation stage which was the 3^rd^ week of March for both years. Local farmers apply pesticides repeatedly on a weekly basis because no action thresholds have been developed against these pests in Pakistan so far [55]. Applications were continued until the second week of May in 2014 and third week of April in 2015, respectively, depending upon pest presence. Spraying was performed using standard Knapsack sprayers ensuring no cross-mixing between extracts. Overall, 9 sprays in 2014 and 6 in the 2015 season were required based on the pest presence in the experimental fields.

### NSE preparation and quantification of azadirachtin A

NSE preparation followed the procedure of Boursier et al. [26]. A 100 g of neem seeds were air dried and depulped, then powdered in an electric grinder (Moulinex, Model 276), subsequently tied in a muslin cloth, and soaked in 1L water for seven days to yield an aqueous extract. For field application, the extract was diluted to a working concentration of 2% (20 mL/L). Thin-layer Chromatography (TLC) and Fourier transform infrared spectroscopy (FTIR) was used to quantify azadirachtin A. Silica-coated TLC plates of 20 x 20 cm were used for thin layer chromatography by using different compositions (1:1) of the mobile phases i.e. diethyl ether: methanol, dichloromethane: acetone, diethyl ether: acetone, isopropanol: n-hexane, dichloromethane: methanol, and dichloromethane: methanol acetic acid. NSE spotted TLC plates were submerged in the respective mobile phases, and ascending movement was observed after covering the TLC plates. TLC plates were removed even after covering them with ¾ parts by mobile phase and drying in a hot air oven for 30 min. Spot formation was visualized under UV visible light after using the different reagents and the R*f* value was determined using the following formula:

$$R_{f}=\frac{D_{solute}}{D_{solvent}}$$

Where *D_solute_* denotes the distance travelled by the solute and *D_solvent_* is the distance travelled by the solvent (mobile phase).

Previously optimized mobile phase (diethyl ether: methanol) from TLC was used as a solvent for the Fourier transform infrared spectroscopy (FTIR) studies by preparing different concentrations of NSE (pure extract, 100, 50, 25, 12.5, 6.25 ppm) which was obtained by using Bruker Alpha ATR-FTIR spectrophotometer (USA). Unknown functional groups and characterizing covalent bonding interactions were observed in spectra in the mid-ranges of wavenumber (4000–500 cm^-1^). The peak area of the respective peaks of different functional groups was calculated from FTIR spectra and plotted against different concentrations to obtain the standard curve. The concentration of azadirachtin A in NSE was calculated from the following equation:

y=mx+b

Where y is the absorbance, m is the slope, x is the concentration, and b is the y-intercept.

### Arthropod sampling, fruit grading and marketability assessments

Sampling, which was performed twice a week in the morning (every 1st and 5th day of a week), started in mid-March until the last week of May in both years. The number of caterpillars was counted from fruits, flowers and leaves by randomly selecting five plants per replicate per treatment. The same selected plants were also assessed for fruit quality and damage. All the fruits from the selected plant were counted, recorded, and sorted as damaged or healthy based on aesthetic value and injury. Harvesting was done following the normal tomato cropping practices and matured tomatoes were manually picked from April through May on multiple occasions. Fruits were visualized for aesthetic value and insect injury. The fruits that were well developed, well formed, free from decay or injury, or had recovered from injury were deemed marketable [47, 56]. Fruits were deemed unmarketable if injury or feeding scars persisted by the time of final harvest. These quality standards have been adopted from previous research [57, 58]. Damaged and undamaged fruits from insecticidal treatments were separated, sorted, and weighed on kg/replicate basis.

### Statistical analysis

The cumulative abundance of *H*. *armigera* and *S*. *exigua* was computed to assess the impact of both insecticides and plant cultivar. A two-way analysis of variance (ANOVA) was used to analyze insecticides and cultivar impacts on *H*. *armigera* and *S*. *exigua* as factorial randomized complete block design experiments, fitting insecticides, cultivar and their interaction as fixed effects and the seasonal pest totals as the dependent response. The significant cultivar-by-insecticide interaction indicated that insecticide effect varied for each cultivar. Further analysis was done to show insecticide effects within each cultivar using repeated measures ANOVA, fitting insecticide, sampling date, and their interaction as independent factors, and pest counts as a dependent response. As these pests were primarily responsible for causing fruit injury, repeated measures ANOVA were run to assess insecticide effects on weekly injured and healthy fruit counts for each cultivar over the entire season. Counts data were log (x+1) transformed to improve compliance with the assumptions of normality and homogeneity of variance. Within each cultivar, the seasonal counts of *H*. *armigera* and *S*. *exigua* were compared across fruit, flowers and leaves using a Chi-Square (χ2) test.

For the final harvest, mean weights of fruits within each category (injured, recovered, marketable and unmarketable) were compared among insecticides by using one-way ANOVA at a 5% level of significance, followed by a Least Significance Difference (LSD; *P*< 0.05) test for mean comparison. These data were analyzed separately between years and cultivars. All analyses were performed in SPSS (version 21) [59].

### Economic analysis

Pest control cost was estimated on a per-hectare basis. The emamectin benzoate purchase cost estimation was based on a market survey performed across the Punjab region of Pakistan. We used retailer price per liter which is tagged on packing and is maintained by regulatory authorities of Punjab province. Pesticide dealers cannot sell the pesticide above this price. The pesticide purchase cost for emamectin benzoate (494 mL/ha) was 12.35 US$/ha. The NSE cost estimation considered the average labour cost for neem seed collection per person per day. When seeds have ripened they fall down on the ground, which can be collected easily and stored in gunny bags under shade for future use. The extract preparation cost used for NSE was 6 US$/ha covering labour charges for collecting, drying and gridding, and the cost of equipment used for simple extraction. The application cost was set at 6 US$/ha. Multiplying purchase cost by application cost gave the pest control cost. The net profit for each treatment was calculated by subtracting the market price from input costs (labour, materials and insecticide application). The economic analysis included the farmer’s personal cost, i.e., the production cost (which does not include insecticide application cost) to grow a hectare of tomato, seed cost for nursery sowing (250 g/ha) = 250 US$/ha; cost of fertilizer, irrigation, labour and nursery raising = 10 US$/ha; cost of land preparation and nursery transplantation = 20 US$/ha, fertilizer cost from transplantation till harvesting = 245.76 US$/ha; labour cost for hoeing = 29.64 US$/ha; labour cost for tomato picking at the time of harvest = 29.64 US$/ha. These price estimations were based on a tomato grower survey conducted across the Multan and Muzaffargarh districts of Punjab, Pakistan. Tomato prices were averaged over the two study years and based on the grower’s actual receipts for those years. The average price per carton was US$ 2.5 and the mean weight was 13 kg. Gross revenue calculation considered an expected yield of 832 cartons/ha multiplied by percent yield and the average price per carton. Subtracting gross revenue from the totals spent on production, chemical purchase and application costs gave net revenue [11, 60]. Cost:benefit ratio (CBR) of each treatment was determined by subtracting the income of the control treatment from the net income of each sprayed treatment and dividing the products by the total cost of plant protection for each treatment [11].

## Results

### Quantification of azadirachtin A in NSE

**Fig 1:**
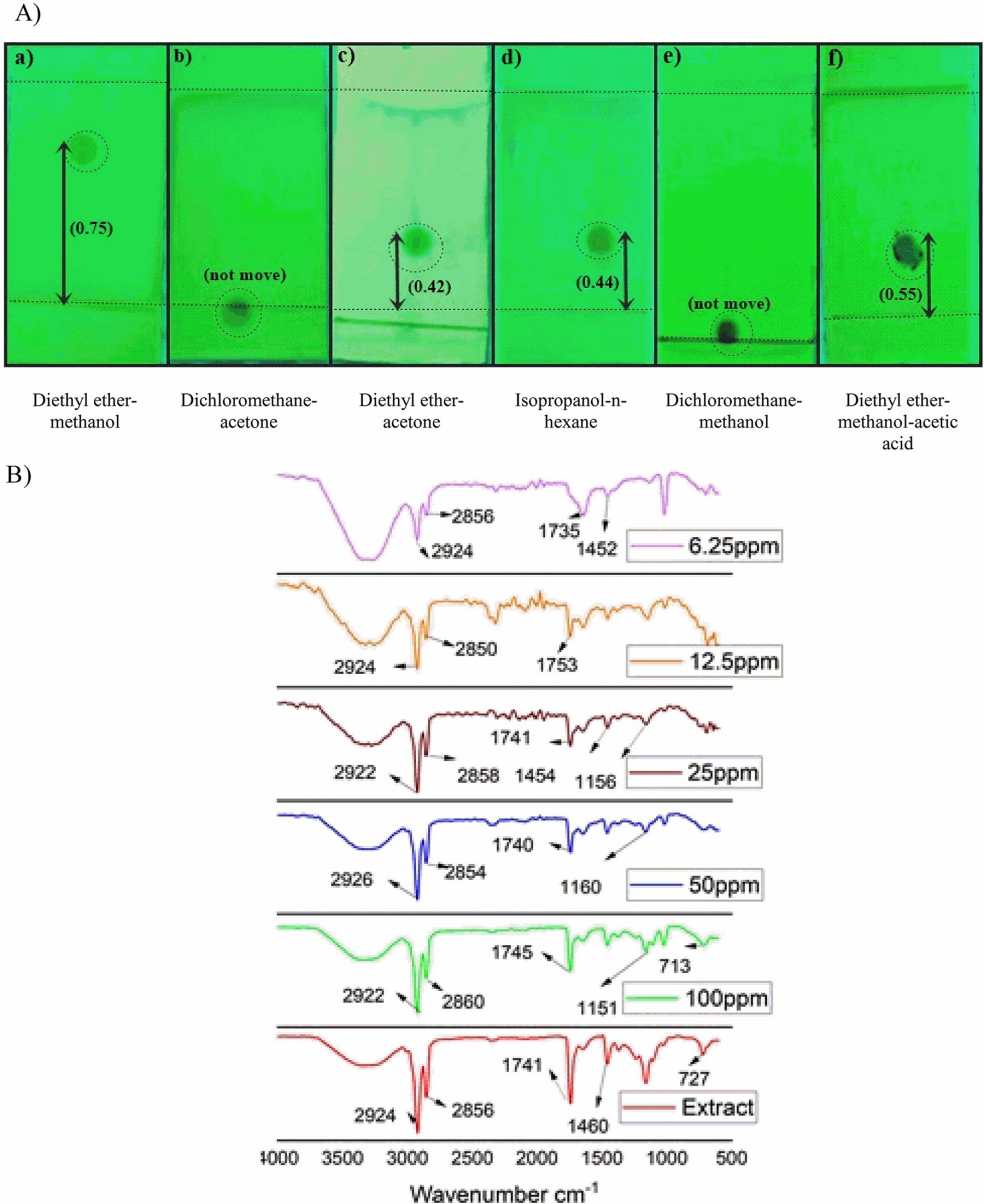
Procedure used for quantification of azadirachtin A in the neem seed extract, where panel (a) represents TLC on silica gel and spot was assessed under UV light in different combination of mobile phase, and (b) represent FTIR spectra of azadirachtin in neem seed extract.

The spot movement and R_f_ value in different ratios of mobile phase were used to choose the appropriate solvent system for purification and quantification of azadirachtin in neem extract (Fig 1). The best diethyl ether-methanol (49:1) solvent system was used for purification of neem seed extract as azadirachtin A moves on TLC plate to an R_f_ value (0.75), while in diethyl ether-acetone (2:1), diethyl ether-methanol-acetic acid (95:5:1), isopropanol-n-hexane (11:9) have R_f_ value 0.42, 0.55 and 0.44, respectively (S1 Table).

**Fig 2:**
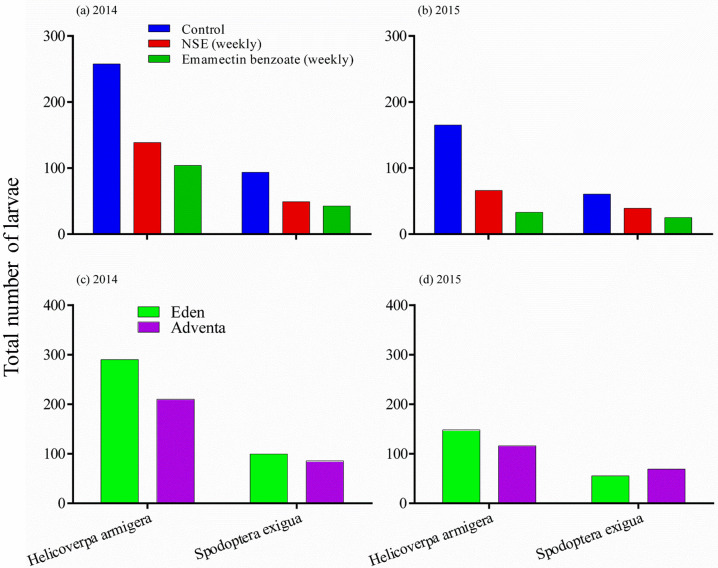
Effects of insecticide treatments (a-b) and tomato cultivars (c-d) on total number of *Helicoverpa armigera* and *Spodoptera exigua*. Fifteen plants were sampled per visit per treatment. Numbers of sampling visits was 9 in 2014 and 6 in 2015. Note differing Y-axis scales.

FTIR spectra of different concentrations of azadirachtin A in neem seed extract are given in Fig 2. In IR spectra a peak was observed at 2854-2920cm^-1^, showing the presence of aliphatic C-H stretching. The C = O stretching of triglyceride ester appeared at 1746cm^-1^ and C-H bending at 1462cm^-1^. The presence of ester was observed at 1164 cm^-1^ which was expected for C-O-C stretching vibration and at 715–723 cm^-1^ was methylene vibration present in the azadirachtin structure. From the FTIR spectra of azadirachtin, the peak area was calculated for each functional group. The wave number and concentration of azadirachtin (S2 Table) was calculated from the standard curve equation obtained from the linearity curve by plotting graphs against peak area and concentration. The concentration of C-H aliphatic, C-H aliphatic, C = O, C-H bending, C-O-C stretching and CH_3_ were 27.4 ppm, 46.2 ppm, 37.7 ppm, 20 ppm, 14.4 ppm, and 14.5 ppm, respectively. The average concentration obtained which indicates the quantity of azadirachtin A in NSE was 26.5 ppm.

### Pest abundance

*Helicoverpa armigera* was more abundant than *S*. *exigua*, and both pests were more abundant on fruits than flowers and leaves for both cultivars (all P< 0.001; Table 1). Table 2 shows the effects of insecticide, cultivar and their interaction on the seasonal sums of *H*. *armigera* and *S*. *exigua* in the 2014 and 2015 growing seasons. The effects were significant for *H*. *armigera* in both seasons. Insecticide impacts were consistently significant for *S*. *exigua* in both seasons, but cultivar impacts were inconsistent and were significant only in the 2015 season. The effect of the cultivar by treatment interaction was frequently non significant for *S*. *exigua*. More larvae were recorded from plots that were unsprayed (Fig 2A and 2B) and from cultivar Eden than Adventa (Fig 2C and 2D).

**Table 1:** Total numbers of *Helicoverpa armigera* and *Spodoptera exigua* larvae on three plant structures of Eden and Adventa cultivars throughout the fruiting stage.

Plant structures	*H*. *armigera*				*S*. *exigua*			
	Eden		Adventa		Eden		Adventa	
	2014	2015	2014	2015	2014	2015	2014	2015
Leaves	9 (4)	8 (7)	11 (6)	1 (1)	4(4)	4 (8)	3 (5)	2(4)
Flowers	43 (16)	15 (13)	11 (6)	5 (6)	23 (24)	5 (9)	2 (3)	9 (16)
Fruit	213 (80)	91 (80)	154 (88)	75 (93)	71 (72)	44 (83)	58 (92)	46 (80)
Total	265	114	176	81	98	53	63	57
Statistics								
*Wald-X* ^*2*^	164.48	77.08	134.06	51.37	48.02	38.74	45.12	35.91
*P*	<0.001	<0.001	<0.001	<0.001	<0.001	<0.001	<0.001	<0.001

Degrees of freedom (*df*) is 2 for all tests. Values in parenthesis are proportion of total population for each respective part

**Table 2:** ANOVA of the effects of insecticides, cultivar, and their interaction on seasonal pest totals in the field over two growing seasons (2014–2015).

	*Helicoverpa armigera*						*Spodoptera exigua*					
	2014			2015			2014			2015		
ANOVAs	*F*	*df*	*P*	*F*	*df*	*P*	*F*	*df*	*P*	*F*	*df*	*P*
Insecticide (I)	215.78	2,12	**<0.001**	959.26	2,12	**<0.001**	29.71	2,12	**<0.001**	86.58	2,12	**<0.001**
Cultivar (V)	72.09	1,12	**<0.001**	72.60	1,12	**<0.001**	3.04	1,12	0.107	16.33	1,12	**0.002**
I × V	11.07	2,12	**0.002**	6.20	2,12	**0.014**	3.2	2,12	0.077	0.58	2,12	0.57

Numbers highlighted in bold indicate significant differences

### Insecticide impacts on weekly pest abundance and fruit injury

**Fig 3:**
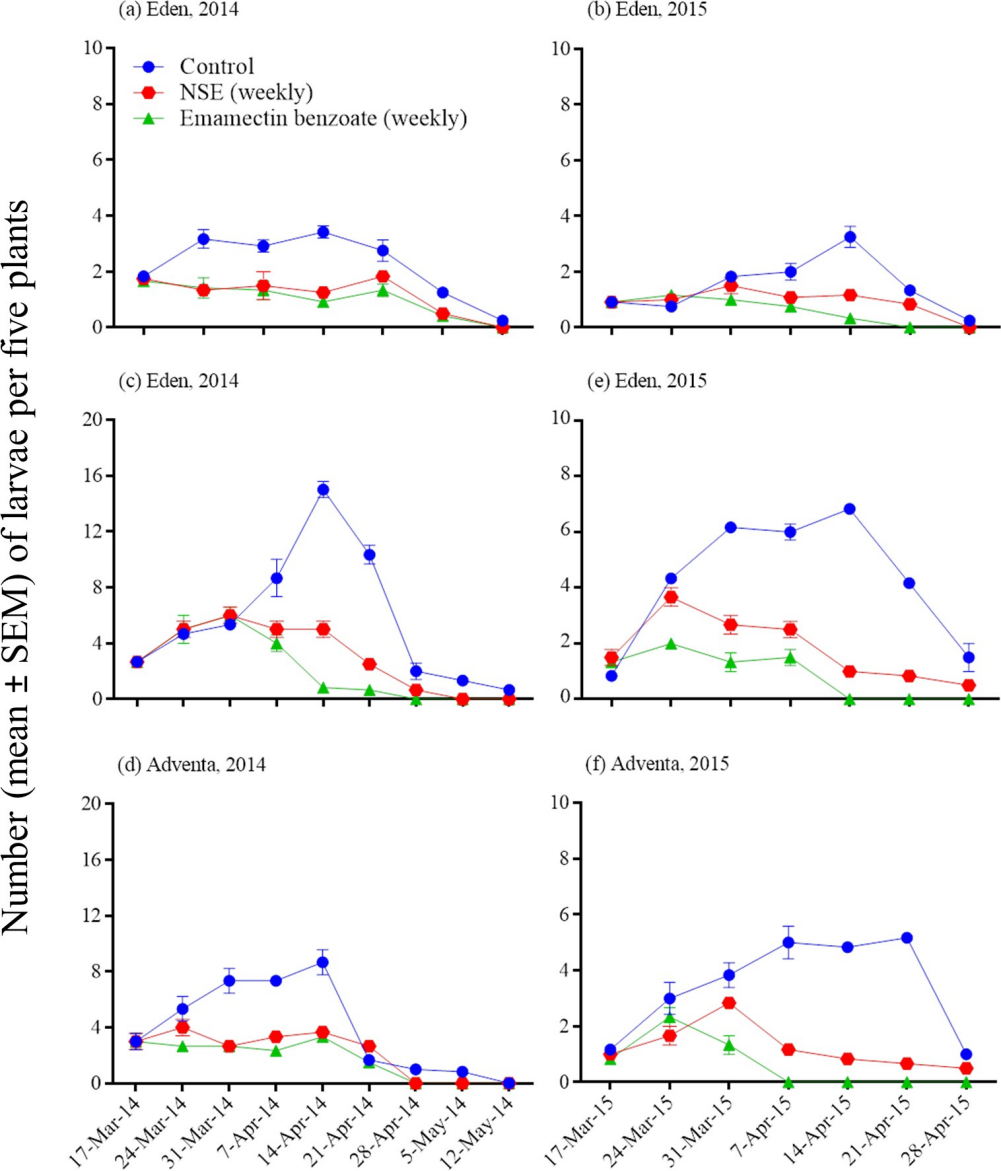
Effects of insecticidal treatments on weekly abundance of *Spodoptera exigua* (a-b) and *Helicoverpa armigera* (c-f) in Eden and Adventa cultivars. Note differing Y-axis scales.

**Fig 4:**
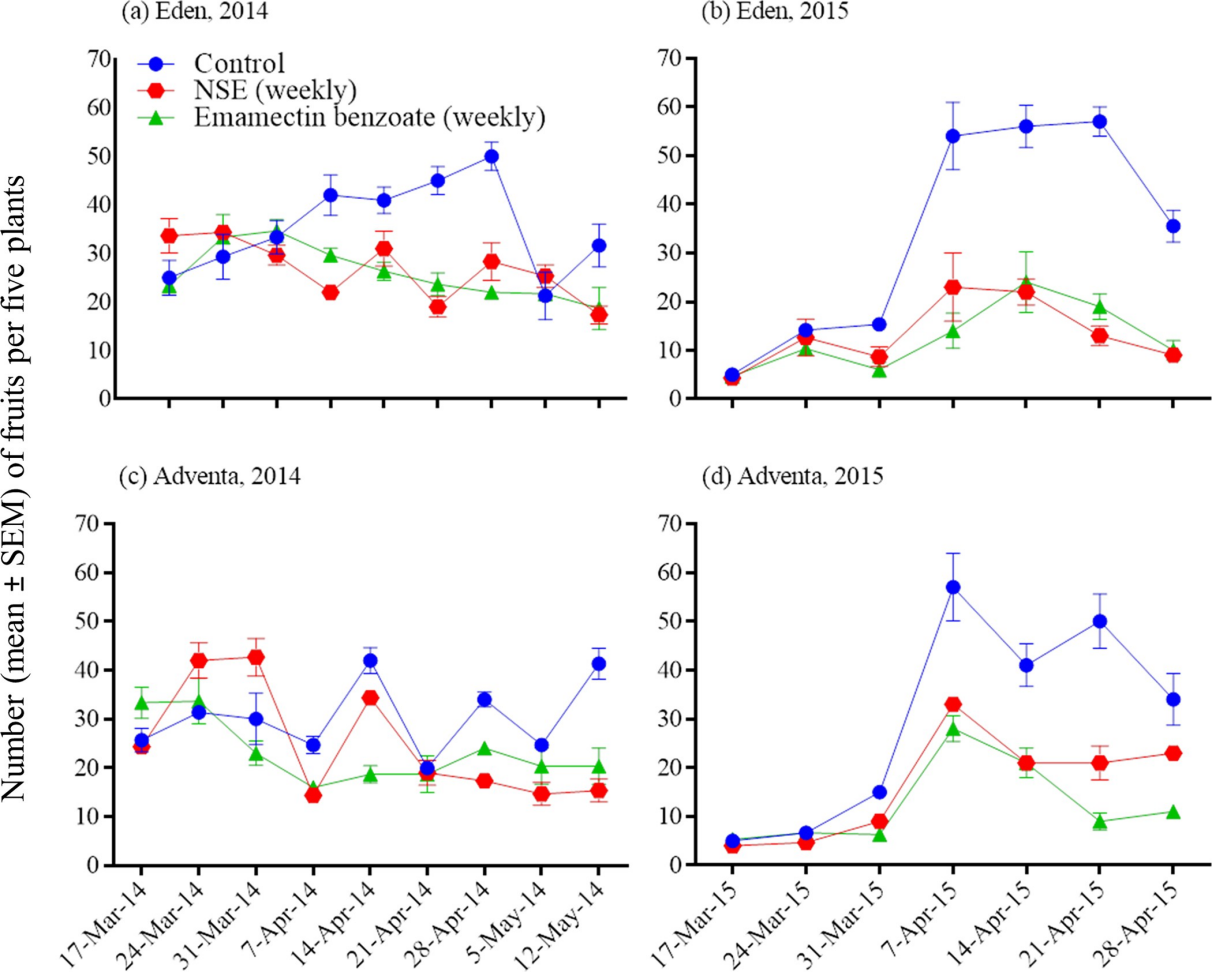
Effects of insecticidal treatments on weekly injured fruit counts (unripe) in Eden and Adventa cultivars.

**Fig 5:**
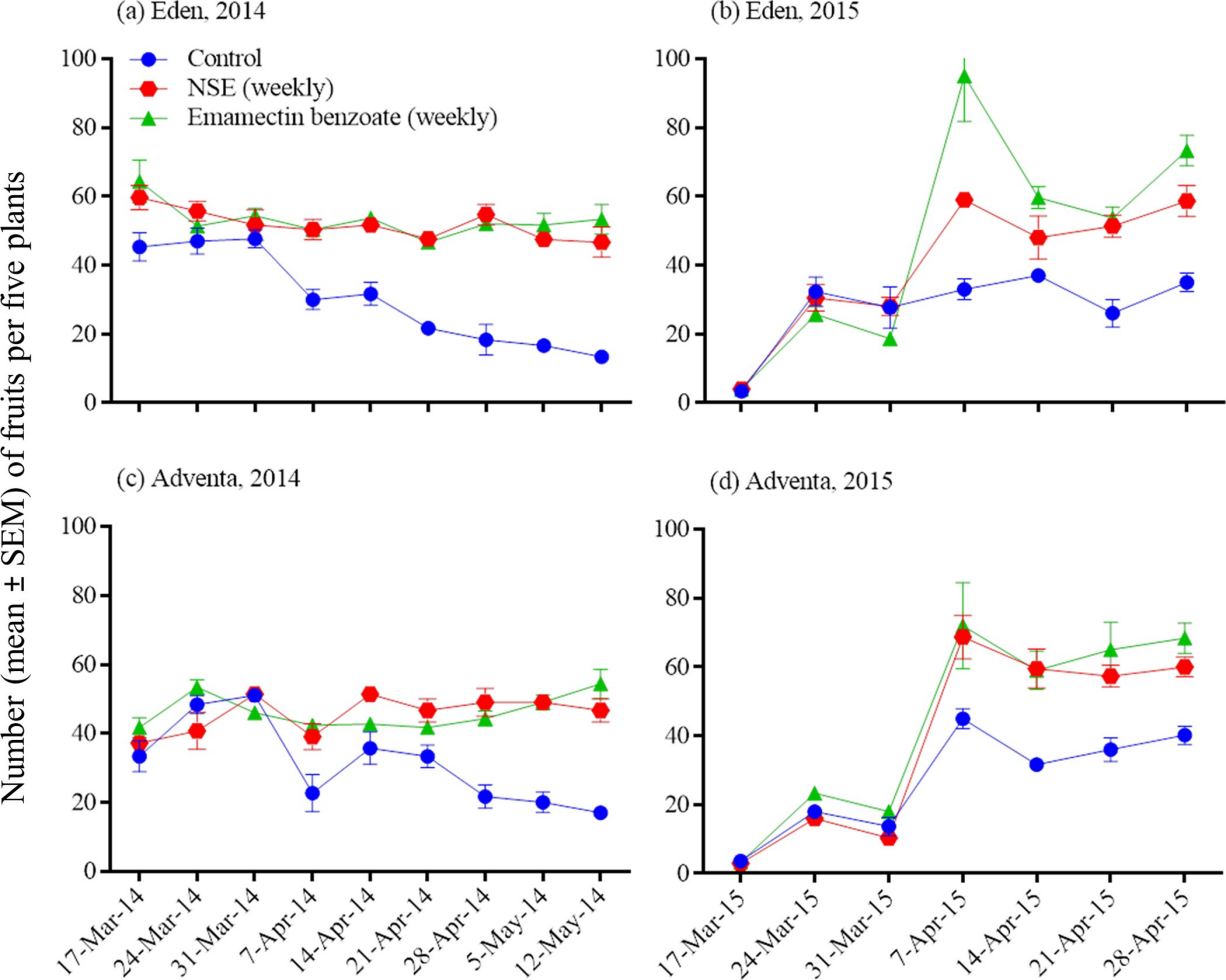
Effect of insecticidal treatments on weekly healthy fruit counts (unripe) in Eden and Adventa cultivars.

Insecticide, sampling date, and their interaction had significant effects on *H*. *armigera* and *S*. *exigua* weekly numbers and injured or healthy fruit counts (repeated measures ANOVA; Table 3; Figs 3–5). The number of pest infestations and injured fruits increased and healthy fruits decreased in the untreated control. Insecticidal treatments over the season caused the pest infestations and fruit injuries to decrease and the healthy fruits to increase but these changes varied with inconsistencies with respect to cultivars and years (Figs 3–5).

**Table 3:** Repeated measures ANOVA of the effects of insecticides, cultivar, and their interaction on weekly pest, injured and healthy fruits counts.

Insect	Variety	Year	Insecticide			Sampling date			Insecticide × Sampling date		
			*F*	*df*	*P*	*F*	*df*	*P*	*F*	*df*	*P*
*Helicoverpa armigera*	Eden	2014	145.90	2,6	**<0.001**	112.23	8,48	**<0.001**	22.41	16,48	**<0.001**
	Eden	2015	340.74	2,6	**<0.001**	69.49	6,36	**<0.001**	27.25	12,36	**<0.001**
	Adventa	2014	46.45	2,6	**<0.001**	109.57	8,48	**<0.001**	8.27	16,48	**<0.001**
	Adventa	2015	445.73	2,6	**<0.001**	30.53	6,36	**<0.001**	21.55	12,36	**<0.001**
*Spodoptera exigua* *	-	2014	19.44	2,6	**<0.001**	24.90	6,36	**<0.001**	2.93	12,36	0.055^NS^
	-	2015	42.80	2,6	**<0.001**	30.52	6,36	**<0.001**	11.03	12,36	**<0.001**
Injured fruits	Eden	2014	3.21	2,6	0.113^NS^	5.35	8,48	**0.013**	5.29	16,48	**0.005**
	Eden	2015	91.66	2,6	**<0.001**	34.81	6,36	**<0.001**	8.80	12,36	**<0.001**
	Adventa	2014	16.17	2,6	**0.004**	15.94	8,48	**<0.001**	8.37	16,48	**0.001**
	Adventa	2015	77.81	2,6	**<0.001**	62.01	6,12	**<0.001**	8.12	12,36	**0.004**
Healthy fruits	Eden	2014	116.62	2,6	**<0.001**	13.22	8,48	**<0.001**	4.71	16,48	**0.007**
	Eden	2015	65.49	2,6	**<0.001**	59.46	6,36	**<0.001**	8.67	12,36	**<0.001**
	Adventa	2014	93.67	2,6	**<0.001**	7.24	8,48	**0.001**	7.10	16,48	**<0.001**
	Adventa	2015	38.11	2,6	**<0.001**	83.55	6,36	**0.003**	3.13	12,36	0.052^NS^

NS denotes non significant difference

* Data were pooled across varieties

Numbers highlighted in bold indicate significant effects

Table 4 presents seasonal means of pests and injured and healthy fruit counts from insecticidal treatments in Eden and Adventa cultivars. Emamectin benzoate suppressed *H*. *armigera* better than NSE in cultivar Eden in both the seasons and in cultivar Adventa only in the 2015 season, whereas this holds true against *S*. *exigua* only in the 2015 season (pooled data across varieties). Injured fruit count was similar between NSE and emamectin benzoate treatments in cultivar Eden during both seasons and in cultivar Adventa only in the 2014 season. Healthy fruit counts were similar between NSE and emamectin benzoate treatments in Eden and Adventa cultivars in 2014, whereas fruit count was significantly decreased in NSE in the 2015 season when compared to emamectin benzoate.

**Table 4:** Seasonal means (per five plants ±SEM) of pests (*Helicoverpa armigera* and *Spodoptera exigua)* and injured and healthy fruits in Eden and Adventa cultivars after insecticidal treatments.

		*H*. *armigera*		*S*. *exigua*†	Injured fruits		Healthy fruit	
		Eden	Adventa		Eden	Adventa	Eden	Adventa
Years	Treatments	Mean ±SE	Mean ±SE	Mean ±SE	Mean ±SE	Mean ±SE	Mean ±SE	Mean ±SE
2014	Control	5.6 ±0.2a	3.9 ±0.2a	2.2 ±0.0a	35.0 ±0.9 a	30.4 ±0.3a	30.2 ±1.6b	31.4 ±0.8b
	NSE (weekly)	3.0 ±0.1b	2.1 ±0.1b	1.2 ±0.1b	26.7 ±0.3b	24.9 ±1.6b	51.7 ±1.1a	45.6 ±1.2a
	Emamectin benzoate (weekly)	2.1 ±0.1c	1.7 ±0.2b	1.0 ±0.2b	25.9 ±0.4b	23.1 ±0.4b	53.1 ±0.8a	46.1 ±0.4a
2015	Control	4.0 ±0.1a	3.3 ±0.2a	1.5 ±0.1a	33.9 ±1.1a	29.8 ±1.6a	27.8 ±1.5c	26.9 ±1.4c
	NSE (weekly)	1.8 ±0.2b	1.3 ±0.1b	0.9 ±0.1b	13.2 ±1.6b	16.5 ±0.7b	39.9 ±0.6b	39.3 ±1.0b
	Emamectin benzoate (weekly)	1.0 ±0.1c	0.8 ±0.1c	0.6 ±0.0c	12.6 ±0.9b	12.5±0.1c	47.1 ±1.4a	44.1 ±1.8a

† Because effects of varieties were non significant on *S*. *exigua* in either year (Table 2), data were thus pooled across varieties for assessing treatment effects. In columns, means labelled with different letters within a year are showing significant differences (LSD test; *P*<0.05) among insecticide treatments.

### Marketability, quality, and cost:benefit ratio

Distinguishing between fruit damage from the two pests was not possible. Since *H*. *armigera* was more abundant than *S*. *exigua*, we assumed the fruit damage to mainly come from *H*. *armigera* infestation. Emamectin benzoate and NSE were statistically similar (P> 0.05) regarding fewer injured fruits, more healed fruits, and not injured fruits in both the Eden and Adventa cultivars. The untreated control treatment had the lowest yield and all poor-quality standards (Table 5). Table 6 presents information on the cost:benefit ratio (CBR) of using NSE and emamectin benzoate in tomato pest management. NSE generated the highest CBR of 5.01 on cultivar Eden and 4.83 on cultivar Adventa in 2014. The following year, NSE generated the best CBR of 9.26 on the cultivar Eden and 7.65 on the cultivar Adventa. Emamectin benzoate generated a CBR of 1.88 on cultivar Adventa and 1.73 on cultivar Eden in 2014. In 2015, emamectin benzoate generated the best CBR of 3.23 on the cultivar Eden and 2.78 on the cultivar Adventa.

**Table 5:** Mean weights (kg/replicate/treatment) of injured, damaged, and marketable fruit yield from insecticidal treatments in Eden and Adventa cultivars.

		Injured but not healed		Injured but well healed		No injury		Marketable yield		
Years	Treatments	Eden	Adventa	Eden	Adventa	Eden	Adventa	Eden	Adventa	
		Mean ± SE	Mean ± SE	Mean ± SE	Mean ± SE	Mean ± SE	Mean ± SE	Mean ± SE	Mean ± SE	
2014	Control	21.6 ± 0.5a	21.5 ± 0.2a	1.5 ± 0.1b	1.3 ± 0.0b	8.9 ± 0.4c	8.6 ± 0.3c	10.4± 0.5b	9.9 ± 0.2b	
	NSE (weekly)	3.8 ± 0.2b	3.8 ± 0.1b	2.8 ± 0.1a	2.9 ± 0.2a	25.7 ± 0.2ab	25.4 ± 0.3b	28.5 ± 0.3a	28.3 ± 0.4a	
	Emamectin benzoate (weekly)	3.6 ± 0.2b	3.8 ± 0.2b	2.6 ± 0.2a	2.1 ± 0.1ab	28.0 ± 0.7a	28.0 ± 0.3ab	30.5 ± 0.6a	30.5 ± 0.5a	
	Mean	9.7 ± 6.0	9.7± 5.9	2.3 ± 0.4	2.2 ± 0.5	20.8 ± 6.0	20.7 ± 6.1	23.1 ± 6.4	22.9 ± 6.5	
2015	Control	21.9 ± 0.3a	22.2 ± 0.1a	2.2 ± 0.3a	1.6 ± 0.2c	12.3 ± 0.3c	10.8 ± 0.4d	9.4 ± 0.1c	12.8 ± 0.5d	
	NSE (weekly)	3.9 ± 0.1b	3.7 ± 0.2b	3.2 ± 0.1ab	3.4 ± 0.1a	25.6 ± 0.3b	25.5 ± 0.3b	28.8 ± 0.2b	28.8 ± 0.3b	
	Emamectin benzoate (weekly)	4.1 ± 0.1b	3.8± 0.2b	2.6 ± 0.1a	3.8 ± 0.1ab	28.5 ± 0.2a	28.2 ± 0.3ab	31.1 ± 0.2a	32.0 ± 0.4a	
	Mean	10.0±6.0	9.9 ± 6.1	2.7 ± 0.3	3.1 ± 0.5	22.1 ± 5.0	21.5 ± 5.4	24.8 ± 5.2	24.6 ± 5.9	

Means in columns in a year labelled with different lower case letters are significantly different (LSD test; *P*<0.05).

**Table 6:** Cost:benefit analysis of using emamectin benzoate and neem seed extract in managing *Helicoverpa armigera* and S*podoptera exigua* on Eden and Adventa cultivars in 2014 and 2015 tomato growing seasons.

Year	Variety	Treatments	Marketable yield (Cartoon/ha)	Number of applications	Pest control cost ($ ha−1)^a^	Gross income ($ ha−1)^b^	Net income ($ ha−1)^c^	Cost:benefit ratio^d^
2014	Eden	Control	150	-	-	376	-209	-
		NSE (weekly)	461	9	60	1155	510	5.01
		Emamectin benzoate (weekly)	498	9	165	1245	495	1.73
	Adventa	Control	145	-	-	364	-221	-
		NSE (weekly)	462	9	60	1156	511	4.83
		Emamectin benzoate (weekly)	513	9	165	1282	532	1.88
2015	Eden	Control	166	-	-	416	-169	-
		NSE (weekly)	457	6	38	1144	521	9.26
		Emamectin benzoate (weekly)	488	6	110	1220	525	3.23
	Adventa	Control	145	-	-	364	-221	-
		NSE (weekly)	454	6	38	1135	512	7.65
		Emamectin benzoate (weekly)	488	6	110	1222	527	2.78

^a^ Insecticide purchase costs are as follows: Emamectin benzoate(494 ml/ha) = US$12.35 per spray; Neem seed collection was charged at 6 US$/ha for season long and the preparation cost per spray was 6 US$/ha. Application cost was charged at 6 US$/ha per spray. Pest control cost was the sum of insecticide purchase cost and application cost.

^b^ Gross revenue ($)/ha = Marketable yield (cartoons/ha) *US$ 2.5 (3-yr average of price per cartoon from whole sale market in 2014–2015).

^c^ Net income was calculated as by subtracting gross income to pest control cost and production cost (a cost of total sum of 585 US$/ha spent on seed and fertilizer purchase and labor cost).

^d^ Cost benefit ratio = Subtracting net income of sprayed treatment to gross income of control treatment and dividing the resulting value by pest control cost of the sprayed treatment.

(-) = not calculable

## Discussion

This research intends to provide baseline data that may be used to develop IPM guidelines for tomato growers to manage *H*. *armigera*. The dominance of *H*. *armigera* over *S*. *exigua* might be due to its high reproductive rate, longevity, larvae survival rates [61], and extensive host range [62]. We find that fruiting stage is the most vulnerable to larval infestation due to the larval preference for fruits over flowers and leaves at the fruiting stage. Jallow et al. [63] and Kakimoto et al. [64] also showed that larvae of *H*. *armigera* prefer fruits and flowers of tomatoes to leaves, stems, and floral buds. From the findings of the present research, we recommend sampling of flowers and fruits of the tomato should be carried out to apply chemicals. It will save time and effort for the growers, however, further research will be needed to determine whether lower populations of lepidopterans on leaves are important in subsequent population development or needs control interventions to avoid yield losses in the latter stages. It has previously been established that larvae of *H*. *armigera* from the third instar onwards are known to be voracious feeders and therefore more destructive. But the first and second instar larvae establish their feeding on the leaves of their hosts and may cause immaterial damage [65].

The cultivars tested here are those widely cultivated in the study area and no previous research reports their potential for conventional host plant resistance. Eden and Adventa cultivars tested in this study affected densities of *H*. *armigera*. Indeed, more numbers of larvae were observed on the Eden cultivar as compared to Adventa. The significant insecticide by cultivar interaction obtained only for *H*. *armigera* but not for *S*. *exigua* suggests the interactive nature of plants, herbivores and pesticides in determining crop losses and controlling the target pests. Phytoalexins are phenolic compounds that have been elucidated recently to induce host plant resistance. When fed on by insects, these phenolic compounds can create further resistance in plants. While we did not evaluate profile of phytoalexins in any of the used cultivars, lower larval densities on the Adventa cultivar compared to Eden suggests that Adventa cultivar might induce resistance against *H*. *armigera* from phytoalexins activities. This kind of complex interaction between plants and herbivores needs to be ruled out carefully in further research.

In this study, the number of sprays required differed between years, depending on the pest presence in the experimental fields. The average temperature in 2014 was lower than 2015. Plausible reasons for more sprays in 2014 may be due to the lower temperature, as well as the interactive effects of other climate factors influenced by temperature, which increase the time to complete different phenological stages of the pests and tomato crop [66]. Emamectin benzoate was more effective in reducing larval densities than NSE but both applications had similar impacts on healthy fruits and marketable yield production. Azadirachtin interferes with egg laying, moulting, pupation, adult formation, respiration, and consumption [67, 68]. Locally prepared neem botanicals have provided comparable control with synthetics for the lepidopteran pests *Leucinodes orbonalis* Guenee and *Plutella xylostella* L. in Nepal and West Africa [69]. Our result corroborates previous reports wherein neem gum nano-formulation, a novel biopesticide prepared from the neem gum extract, caused 100% antifeedant, larvicidal, and pupicidal activities against *H*. *armigera* and *S*. *litura* [70]. Locally prepared extracts of neem provide effective control due to their novel mode of action and are less toxic biopesticides that are being advocated as alternatives in contemporary pest management [12, 71–74]. Moreover, farm workers and operators will be safer if such biopesticides are adopted, as it is estimated that 25 million people are poisoned by synthetic pesticides from developing countries every year [28].

Economic analysis of NSE used in the current study shows that NSE is a more cost-effective option for smallholder farmers than using synthetic pesticides. The highest cost:benefit ratio results of 1:9.2 were observed for NSE as compared to the cost:benefit ratio 1:3.2 of plots sprayed with emamectin benzoate. Higher cost:benefit ratio has been reported to manage *H*. *armigera* on chickpeas and okra when using *A*. *indica* extract at 5% concentration [15]. Our results also agree with Amoabeng et al. [11] who reported the highest cost:benefit ratio (1:29) observed for plots sprayed with botanicals as compared to the cost:benefit ratio (1:18) observed for plots sprayed with conventional insecticides. In another similar study, extracts of local weeds resulted in economically viable control of several key pests including beetles on beans, which was comparable to that induced by the pyrethroid insecticide lambda-cyhalothrin with a higher marginal rate of return [75]. Neem seed extracts proved to be effective control measures against insect pests of wheat, cabbage and cauliflowers and increased the yield of these crops considerably [14, 19, 25]. Tembo et al. [20] showed that using extracts of plants with insecticidal potential to control pests of legumes can be equally as effective as synthetic insecticides with reference to crop yields.

Composition of azadirachtin and constituents responsible to act as insecticide vary greatly in the neem seeds. Important factors that affect quantities include, among others, neem seeds collected from different geographic regions, timing of collection of seeds, climate, genetic diversity, variations in plant morphological structures and physiology, and storage of neem tree parts [17, 76, 77]. Kaushik et al. [78] and Tomar et al. [79] reported comparable variations in the azadirachtin composition of neem seeds collected from different regions of India. Chemical characterization from collected plant parts is inevitable to get reproducible results. Our research developed NSE and provided necessary information needed for NSE incorporation as pesticide into existing IPM programs. Higher populations of pests in NSE-treated plots but lower feeding injury is attributed to antifeedant and molting disruptor modes of action of azadirachtin. This compound can act as insect antifeedant at the concentration of 1 part per million and no other antifeedant has been reported to be effective at such a low concentration [80, 81]. The concentration of azadirachtin was higher in NSE prepared for our research trials than this lowest concentration required to act as antifeedant.

## Conclusion

*Helicoverpa armigera* and *S*. *exigua* abundance and tomato fruit losses varied among cultivars, insecticides and years/cropping seasons. NSE managed to produce a marketable yield similar to the synthetic counterpart despite harbouring more larvae of *H*. *armigera* and *S*. *exigua* by potentially reduced feeding due to antifeedant activity. The cost:benefit ratio that NSE generated was even higher than that obtained following synthetic pesticide application. Hence, NSE offers promise to make IPM programs more sustainable and economically profitable by reducing synthetic chemical pesticide loads and concerns without sacrificing marketable yields. Thus, NSE prepared from locally available neem trees can be very effective and helpful for small holder farmers in developing countries. It was argued that formulated botanicals are well suited for industrialized countries for organic farming, but locally prepared extracts should be part of IPM programs in developing countries [6, 48]. We also recommend further research on the rotational use of the NSE with synthetic pesticides for better field effectiveness and for practical management of the problem of insecticide resistance.

## Supporting information

S1 Table R_f_ value of azadirachtin in different solvent system by thin layer chromatography. (DOCX)

S2 Table Quantification of azadirachtin in extract by fourier transform infrared spectroscopy (FTIR). (DOCX)
