# Supplementary material for: Botanical biopesticides have an influence on tomato quality through pest control and are cost-effective for farmers in developing countries
Source: PLoS One. 2023 Nov 28;18(11):e0294775. doi: 10.1371/journal.pone.0294775 (PMC10684083; doi:10.1371/journal.pone.0294775)
Supplement: S1 Table — (DOCX) [file pone.0294775.s001.docx]

# S1 Table. R_f_ value of azadirachtin in different solvent system by thin layer chromatography

| **Mobile Phase** | **Ratios** | **R_f_ value** |
| --- | --- | --- |
| Diethyl ether-methanol | (49:1) | 0.75 |
| Dichloromethane-acetone | (4:1) | Not move |
| Diethyl ether-acetone | (2:1) | 0.42 |
| Isopropanol-n-hexane | (11:9) | 0.44 |
| Dichloromethane-methanol | (19:1) | Not move |
| Diethyl ether-methanol-acetic acid | (95:5:1) | 0.55 |
