# Supplementary material for: Botanical biopesticides have an influence on tomato quality through pest control and are cost-effective for farmers in developing countries
Source: PLoS One. 2023 Nov 28;18(11):e0294775. doi: 10.1371/journal.pone.0294775 (PMC10684083; doi:10.1371/journal.pone.0294775)
Supplement: S2 Table — (DOCX) [file pone.0294775.s002.docx]

# S2 Table. Quantification of azadirachtin in extract by fourier transform infrared spectroscopy (FTIR)

| **Functional group** | **Wavenumber (cm^-1^)** | **Y=mx+b** | **Concentration (ppm)** | **Average concentration of Azadirachtin in extract** |
| --- | --- | --- | --- | --- |
| C-H aliphatic | 2924 | y=2.14  m=0.071  b=0.192 | 27.4 |  |
| C-H aliphatic | 2856 | y=2.08  m=0.033  b=0.559 | 46.2 |  |
| C=O | 1743 | y=2.09  m=0.032  b=0.886 | 37.7 | 26.5 |
| C-H bending | 1456 | y=2.03  m=0.075  b=0.537 | 20 |  |
| C-O-C stretching | 1158 | y=2.06  m=0.100  b=0.621 | 14.4 |  |
| CH_3_ | 717 | y=2.02  m=0.124  b=0.226 | 14.5 |  |
